# Efficacy of the use of perfluorocarbon as a temporary tamponade agent in severe ocular trauma and/or complex retinopexy: a scoping review

**DOI:** 10.1186/s40942-023-00504-6

**Published:** 2024-01-18

**Authors:** Sara Margarita Pérez Pérez, Valentina Cárdenas Arias, Daniela Jaramillo Ramírez, Camilo Eduardo Martínez, Nathalie Tamayo Martínez

**Affiliations:** 1 Unidad de Oftalmología. Hospital Universitario San Ignacio. Bogotá, Bogotá, Colombia; 2 Facultad de Medicina, Pontificia Universidad Javeriana. Bogotá, Bogotá, Colombia; 3 Ophthalmology Unit, Pontifical Xavierian University, Street 7 # 40-62, Bogotá, Colombia; 4 Departamento de Epidemiología Clínica, Facultad de Medicina, Pontificia Universidad Javeriana, Bogotá, Colombia

**Keywords:** Perfluorocarbon, Temporary tamponade, Retinal detachment, Severe ocular trauma, Scoping review

## Abstract

**Background:**

Perfluorocarbon (PFC)possesses unique chemical properties that favor the pigment epithelium’s adhesion and allows the drainage of subretinal fluid through retinal holes present in retinal detachment cases. However, PFC as a temporary tamponade agent has been limited due to its high potential for toxicity.

**Main body:**

We conducted a scoping review regarding the use of PFC in vitreoretinal surgery as a temporary tamponade in subjects with severe ocular trauma or severe retinal detachment who received a therapeutic intervention (vitrectomy via posterior approach with the use of PFC as a temporary tamponade), compared to vitrectomy without the use of PFC as a temporary tamponade. Outcomes of interest were retinal reattachment, visual acuity (VA), postoperative complications and retinal toxicity. The search was performed in Medline, Medline In-Process & Other Non-Indexed Citations, Medline Daily Update, Embase databases. Reference lists from relevant review articles were also included. Two hundred thirty-eight studies were found, with no duplicate entries. In the first selection, 230 articles were eliminated; in the second selection, 6 additional articles were discarded. In total, 8 articles were obtained in this review. Two selected articles corresponded to animal studies and 6 to studies in humans. Regarding study design, 5 were case series, and 1 was a cohort study.

**Conclusion:**

PFC as a short-term tamponade had high rates of reapplication, improved VA, and the most frequent adverse effects were reversible after PFC withdrawal. Nonetheless, the quality of the studies was poor. Studies with more rigorous methodologies are needed to determine visual and structural outcomes and potential risks of PFC use as a temporary tamponade in vitreoretinal surgery.

**Supplementary Information:**

The online version contains supplementary material available at 10.1186/s40942-023-00504-6.

## Background

PFC is a family of highly cohesive heavy hydrocarbon compounds with high surface tension. PFC is a synthetic liquid with unique chemical properties that favor its use in multiple settings, especially in vitreoretinal surgery [1]. Highly purified perfluorodecalin, a PFC, has been used in the last decade as an ocular tamponade agent in severe ocular trauma surgery. On one hand, PFCs have a density that is twice the one of water and low viscosity [1, 2]. These properties provide a high cohesion to the inner surface of the eyeball, exerting a significant force on the retina [2, 3] and thus favoring the adhesion of the pigment epithelium in cases of retinal detachment. On the other hand, it allows the drainage of subretinal fluid through the retinal holes present. This could potentially serve as a starting point for further investigation, as PFC may have the potential to ascertain the stability of the retina over a longer duration. Consequently, we undertook the task of conducting a scoping review to systematically gather and analyze all the existing evidence that may support to the notion that PFC holds promise as a short-term tamponade overweighting its potential risk for toxicity.

It is important to note that, PFC use as a short-term tamponade agent has been limited due to its high potential for toxicity specifically perfluorooctane. Stolba et al. found that its use for more than two weeks was associated with direct lesions in the retina, especially in the photoreceptor cell layer, damage that became more severe after the fourth week of exposure [4]. It also appears to be associated with decreased retinal vascular flow [3]. Therefore, PFC is not used on a long-term basis. However, newer highly purified PFC molecules (for example, highly purified perfluorodecalin) are more lipophilic. This property reduces toxicity, favors its complete extraction, and may be helpful as a temporary tamponade to achieve better retinopexy while reducing its harmful effects. Therefore, this study aims to describe retinal reattachment, VA, and adverse effects of PFC as a short-term tamponade agent used in severe ocular trauma or retinal detachment surgery.

## Methods

We conducted a scoping review regarding the use of PFC in vitreoretinal surgery as a short-term tamponade, following the population-intervention-control-outcome (PICO) framework: (P) subjects with severe ocular trauma or severe retinal detachment who underwent vitrectomy via posterior approach; (I) use of PFC as a temporary tamponade; (C) no comparison was necessary as we expected to find few studies some of them without a comparison group; (O), retinal reattachment, VA, postoperative complications and retinal toxicity.

Case series, original articles, cohort studies, and clinical trials in English, Portuguese, or Spanish were included. Articles corresponding to editorials and research protocols were discarded. Animal and human studies were included.

The selection criteria for participants were subjects of any age with severe ocular trauma or severe retinal detachment. The intervention was posterior vitrectomy using PFC as a temporary tamponade. The primary outcomes were retinal reapplication and VA. Secondary outcomes were intraoperative and postoperative complications associated with the use of PFC such as retinal toxicity, cataract, epiretinal membrane, ocular hypertension, and uveitis.

We searched in Medline, Medline In-Process & Other Non-Indexed Citations, Medline Daily Update, and Embase databases from insertion to 21 December 2022. We used free controlled and uncontrolled terms *“*perfluorocarbon”, “eye injury”, “ocular trauma”, and “vitreoretinal surgery”, combined with Boolean operators. We also employed the “snowball” method search for articles from the references of useful articles. The complete search algorithms are described in Supplementary Tables 1 and 2.

**Fig. 1:**
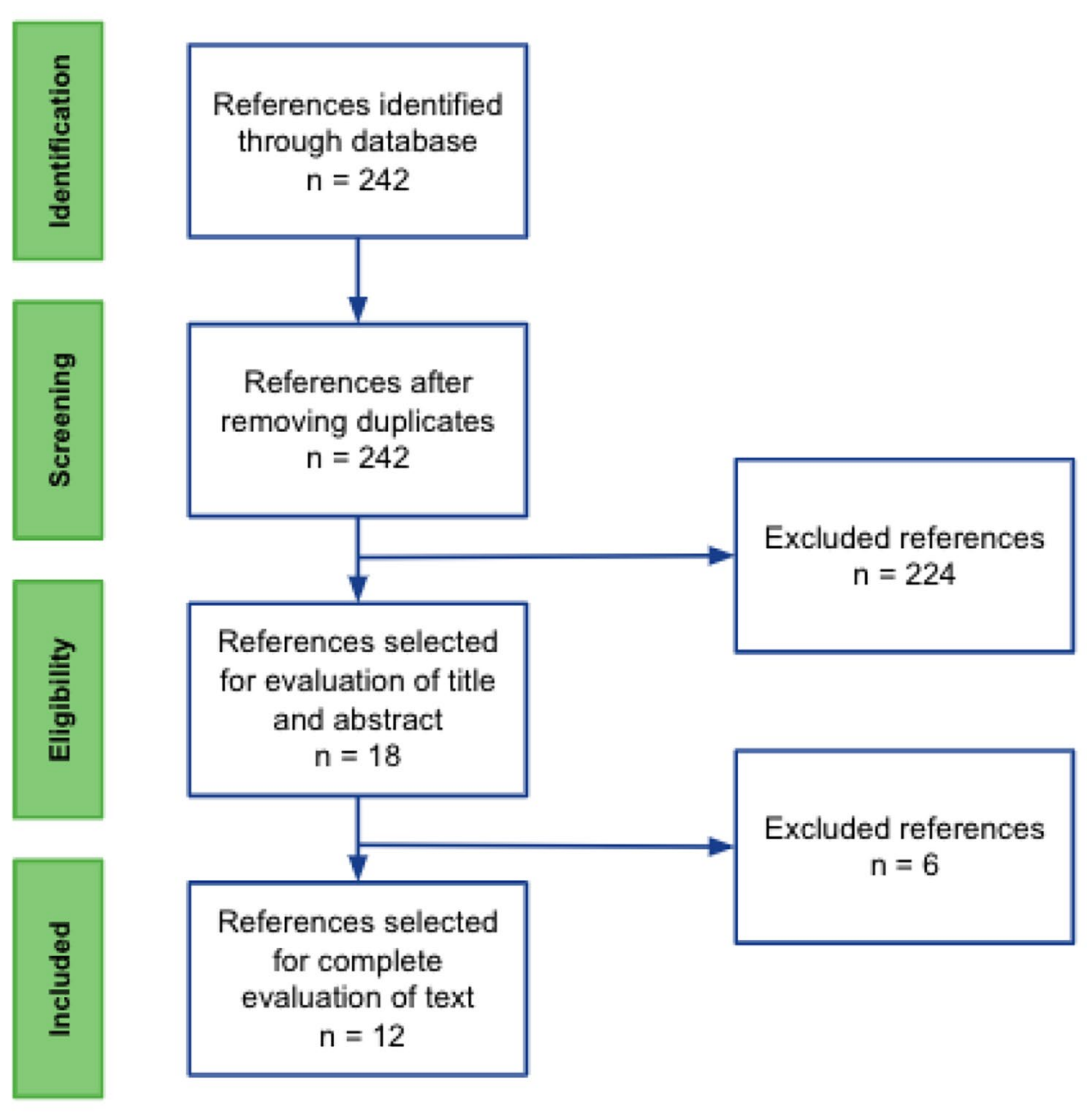
Prisma flow diagram showing selected studies

After eliminating duplicate manuscripts, SP and VC performed an independent selection of the studies. The selection of articles was carried out in two stages. In the first stage, we choose manuscripts based on titles and abstracts. In the second stage, we evaluated full texts and references. The discrepancy between the authors in the selection was reconciled between them, or a third author could intervene. Figure 1 shows the article selection process.

Two authors extracted the data independently. We created a data collection template that included, amongst others: study objectives, study design, period of data collection, ethics committee approval, population characteristics, inclusion criteria, exclusion criteria, recruitment method, number of people in the study, group differences at baseline, characteristics of vitrectomy and PFC application, time of PFC application, VA, retinal reattachment, postoperative complications and retinal toxicity. For the evaluation of possible biases, we used the *Newcastle-Ottawa Scale (NOS)*, a tool that allows the evaluation of biases in case-control and cohort studies.

Cumulative incidences were calculated for each outcome in each study and risk ratios if there was a comparison group for the outcome. If the studies’ clinical outcomes and methodological characteristics are sufficiently similar we would perform a meta-analysis. Otherwise, we make a narrative description of the studies.

## Results

### Selected and included studies

A total of 242 studies were found, with no duplicate entries. The articles are presented according to the guidelines recommended by the *Preferred Reporting Items for Systematic Reviews and Meta-Analyses* (PRISMA) in Fig. 1. In the first selection, 224 articles were eliminated due to reasons related to: inclusion and exclusion criteria, PFC not being use as a short term tamponade, articles corresponding to editorials and research protocols; in the second selection, 6 additional articles were discarded. In total, 12 articles were obtained in this review. Table 1 and Supplementary Table 3 show the characteristics of the selected studies, and Supplementary Table 4 shows the characteristics of those excluded. Two selected articles corresponded to animal studies and 6 to studies in humans. Regarding study design, 5 were case series, and 1 was a cohort study.

**Table 1:** Articles included in the scoping review

Author	Year	Study type	Retinal reattachment N (%)	Complications N (%)
Bhardwaj G. et al. [5]	2020	Case series	19 (100%)	cataract, PFC in anterior chamber, macular pucker (5%)
Maya Eiger-Moscovich et al. [6]	2017	Case series	13 (100%)	ocular hypertension 2 (15%), cataract 2 (15%), cystoid macular edema 2 (15%)
Joseph Pikkel et al. [7]	2013	Cohort study	22 (100%)	cataract 4 (13.6%) ocular hypertension 4 (13.6%) epiretinal membrane 2 (7%)
Marcelo Carvalho VenturaI et al. [8]	2007	Case series	8 (80%)	hipotonía ocular 3 (37.5%)
Rofail, Marc et al. [9]	2005	Case series	15 (93.7%)	cataract 5 (31.5%)
M Sirimaharaj et al. [10]	2005	Case series	58 (93.5%)	cataract 51 (80.5%)
Daminana Zeana et al. [11]	1999	Clinical Animal Experiment	Not evaluated as an outcome	peripheral atrophy of the myelinated nerve fibers (14,5%)
Luca Migliavacca et al. [12]	1998	Clinical Animal Experiment	Not evaluated as an outcome	pits on the inner retina ​​
Drury B, Bourke RD	2009	Case series	76%	superior redetachment, atrophic macular changes, minor macular haemorrhage, cataract, elevated intraocular pressure, corneal defects, PVR, epiretinal membrane and retained perfluorocarbon heavy liquid.
Chehade LK et al.	2021	Case series	98.8%	Cataract formation
Bottoni, F et al.	2013	Case series	9 (82%)	Macular pucker 1 (9%)
Rush R, et al.	2012	Case series	39 (100%)	Capsular opacification 28 (71%)

We evaluated the risk of bias with t*he NOS.* The cohort study [5] is of low quality due to the small number of participants (N = 36). The data were retrieved from the medical records. Subjects exposed corresponded to subjects who underwent vitrectomy after the intervention was implemented in the study site. Subjects unexposed corresponded to subjects who had been treated before the intervention was implemented. Therefore, exposed and unexposed groups are likely to be dissimilar. Additionally, studies were deemed not similar enough to carry out a meta-analysis.

In the studies with a human population, the average age ranged between 40 and 55 years and consisted of predominantly male subjects. All subjects had giant retinal tears and 1 study included patients with ocular trauma. The PFC used was perfluoro-n-octane (PFnO) in two studies. The average application time ranged from two days in animals and 44 months in humans.

#### Complete retinopexy

The prospective case series of Bhardwaj et al. [6] included 19 subjects with recent giant retinal tears who underwent pars plana vitrectomy with PFC injection, transscleral diode laser retinopexy to the edge of the giant retinal tear, and short-term postoperative heavy fluid tamponade for four to eight days. The retinal reattachment rate was 100%, and only 3 subjects required additional surgery.

Eiger-Moscovich et al. [7]. described a retrospective case series of 13 subjects who presented to a tertiary medical center with giant retinal tears between November 2011 and August 2015. Surgery was performed by an experienced retinal surgeon. All retinas were successfully fixed intraoperatively. The retina remained attached in 12 subjects (92%) without additional vitrectomies.

Pikkel et al. [5] in a cohort study, evaluated PFC as a short-term postoperative tamponade in 22 subjects with giant retinal tears. In all subjects, retinas were reattached. In a similar study by Carvalho et al. [8] 10 subjects kept the PFC for five days postoperatively, with 80% of the retinas remained attached during the 16-month follow-up.

Likewise, in the study by Rofail et al. (9 [1]) the retinal reattachment rate was 100% after primary vitrectomy in 16 subjects with giant retinal tears, using perfluorocarbon-n-octane as a tamponade agent. One retina detached (6.3%) and needed reattachment after a primary intervention. Sirimaharaj et al. [9] in their retrospective study of 65 subjects with rhegmatogenous and traumatic retinal detachment, described that at a mean follow-up of 24.5 months, 80.6% of the operated eyes had adequate retinopexy. On the other hand, recurrence of detachment was documented in an average of 26.7 days in 12 of 65 (18.5%) eyes after PFC removal.

In the experimental animal studies, retinopexy was not evaluated as an outcome [10, 11]. These studies focused on safety on the use of PFC as a temporary tamponade.

In the study conducted by Drury et al. [12], Short-term perfluoro-*n*-octane tamponade achieved a stable reattachment rate of 76% when used to manage challenging retinal pathologies.

#### Visual accuity

In the study by Bhardwaj et al. [6] the subjects’ best corrected visual acuity (BCVA) at the end of follow-up was 20/40 or better in 11 eyes (58%), between 20/60 and 20/200 in seven (37%) and 20/400 in 1 (5%). In Eiger-Moscovich’s et al. [6]. study, the macula was detached in 3 of the 13 subjects. However, in all subjects BCVA improved after surgery. The final BCVA was equal to or better than 20/100. Of the 10 subjects in whom the macula was attached at the time of surgery, 6 (60%) had a final BCVA equal to or better than the initial one.

In the study conducted by Pikkel et al. [5] in the group without PFC VA was 6/12 or better in 26.7% of subjects vs. 73.3% in the study group. VA worsened in 22.7% of subjects in the PFC group and in 57.1% in the control group (p = 0.054).

In the series of Carvalho et al. [8]. 50% of the subjects improved the initial preoperative BCVA. Rofail et al. [13]. showed that 11 of the 16 subjects who underwent surgery (68.8%) had improved VA. However, 18.6% of the subjects who underwent surgery showed a deterioration of VA due to macular tears or the development of postoperative epiretinal membranes. Sirimaharaj et al. [9]. found that 54.8% of the operated eyes showed improvement in VA. In 45.2% of them, the improvement was at least two lines on the Snellen chart. Likewise, 12.9% of the eyes presented visual impairment secondary to the development of cataracts, macular compromise related to retinal detachment, and retinal reattachment.

In the series of Chehade et al. [14], they demonstrated a 98.8% anatomical success and significant improvement in BCVA, with no reports of retinal toxicity.

Likewise in the study conducted by Botoni et al. [15], 64% of the reattached eyes had a final visual acuity of 20/40 or better).

#### Postoperative complications

In the study by Bhardwaj et al. [6]. PFC droplets were observed in the anterior chamber in 5 of 20 eyes (2 phakic and 3 aphakic). The droplets were removed at the time of heavy fluid removal from the vitreous cavity. None of these eyes developed corneal decompensation during follow-up. Pikkel et al. [5] found no difference in postoperative complication rates (cataract, epiretinal membrane, ocular hypertension, and uveitis) between the control group and those who received the intervention. No corneal toxic effect was found in the study group, nor hypotension or residual subretinal PFC.

Eiger-Moscovich et al. [6] documented postoperative elevation of intraocular pressure in 2 of 13 subjects which were successfully treated with topical antiglaucoma medications. PFC was also observed in the anterior chamber in 1 of them, requiring paracentesis for its removal. Two subjects developed cataracts, and 2 others developed cystoid macular edema (CME).

Carvalho et al. [8] observed in 3 of 10 subjects ocular hypotony associated with a cellular reaction in the anterior chamber and vitreous cavity (anterior to PFC) in the early postoperative period. Difficulty in fundus visualization was also present in 3 subjects (30%). Rofail et al. [13]. also observed ocular hypotony in 3 of the 16 subjects. One subject had a severe drop in intraocular pressure after removing the tamponade agent and a fibrin-mediated inflammatory reaction in the anterior chamber, which resolved with topical anti-inflammatory agents. In the study by Sirimaharaj et al. [9] 1 of the 65 subjects developed increased intraocular pressure secondary to lens displacement due to the pressure and angular closure exerted by the PFC. Cataracts developed in 80.5% of the operated eyes. Likewise, 3 subjects developed glaucoma, in whom adequate control of intraocular pressure was achieved and the presence of PFC residues in the anterior chamber was ruled out.

As for experimental animal studies, Zeana et al. [10]. found no retinal toxicity at 9 weeks in any rabbit after an intravitreal injection of 1.0-1.2 ml of perfluorohexyloctane. However, Orzalesl et al. [11] evidenced in rabbits exposed to perfluorodecalin for more than 6 days liquid content of PFC in lower retinal segments in relation to the point of greater amount of tamponade, also in the photoreceptor cell layer presenting associated retinal edema generating irreversible damage to the retina.

## Discussion

PFC seems to achieve retinopexy in severe retinal detachment. Nonetheless, its use has been limited by the high potential for toxicity previously described [5]. With this scoping review we summarized evidence on PFC as a temporary tamponade in vitreoretinal surgery. We were interested on evaluating outcomes retinal reattachment, VA, postoperative complications, and potential retinal toxicity. Eight studies met the selection criteria, of which five corresponded to case series, two to experimental animal studies, and 1 to a cohort study. The latter has a high risk of selection and confounding biases because they selected the exposed and un-exposed group at different times.

Retinal reattachment rate ranged from 80 to 100%. For example, the studies by Bhardwaj et al. [6]., Carvalho et al. [8]. and Sirimaharaj et al. [9]. agree that PFC ensures complete retinopexy and a better final VA than preoperative VA. Many studies [16–18] have reported a visual improvement that varied from 56.8 to 60%.

Even if VA might be used as a marker of successful functional recovery of the eye. Still it has its limitations, since VA might change due to other factors such as lens status changes and epiretinal membrane development. Additionally, ocular optical coherence tomography techniques which evaluate preoperative and postoperative anatomical changes might not match with preoperative and postoperative visual states. Currently, no single test has an optimal correlation between anatomical outcomes and VA [19].

Most subjects who underwent posterior vitrectomy using PFC as a tamponade agent had no deterioration in VA. Rofail et al. reported improved VA in 68.8% of subjects, and Pikkel et al. [5] reported improved VA in 50% of subjects in the study group versus improvement in only 14.3% of the control group. VA deterioration was related to macular compromise of their underlying pathology, for example, epiretinal membranes, among other complications unrelated to PFC.

The incidence of adverse events was low in human studies using PFC as a temporary tamponade showed by Ryan et al. [20] that demonstrated that the use of Perfluoro-n-octane as a short-term vitreous substitute in primary rhegmatogenous retinal detachment repair cases involving inferior/multiple breaks or giant retinal tears (GRTs) shown both efficacy and safety. The substantial reduction in its side effect profile can be achieved through Perfluoro-n-octane removal within a 10-day timeframe.

Regarding complications, mainly ocular pressure alterations (hypotony or ocular hypertension) were described. Subjects who developed secondary ocular hypertension were adequately treated with tamponade removal and topical hypotensive medication [8]. These ocular pressure complications were found to be present in less than 50% of subjects in each study. Adverse events reported using PFC in other contexts mention that the occurrence of subretinal retention of liquid PFC bubbles can impact between 1 and 11% of pars plana vitrectomy (PPV) cases conducted for rhegmatogenous retinal detachment (RRD) [21, 22]. The appropriate time for PFC removal remains a subject of ongoing discussion; however, a consensus exists on the importance of prompt and proactive management due to the potential for resulting irreversible visual impairment that may present as a confounding factor in final VA [19].

In the two animal studies, rabbits were used and had opposite results. One of irreversible retinal damage conducted by Orzalesl et al. [11], and the other without retinal toxicity conducted by Zeana et al. [10]. These findings may correspond to the use of different PFC molecules: perfluorohexyloctan, which has a higher concentration of PFC, and perfluorodecalin, respectively. They also differ in the purity level of the compounds.

Studies suggest that PFC is safe and effective for managing complex retinal detachment though a conclusive affirmation cannot be made due to the risk of bias of the studies. Although, the quality of evidence is low, as all studies had small sample sizes; there were differences in the duration of tamponade use, administration intervals, and follow-up, and most did not have a comparison group. The inclusion of studies involving both human subjects and animals certainly presents a potential challenge to the methodological rigor and overall credibility of our analysis., though the rationale behind this approach stems from the aim of maximizing the available evidence. Including animal studies alongside human research allows for a broader perspective and provides insights that may not be accessible through human studies alone.

Universally complex retinal detachment is treated with short-term tamponades such as gas or silicon though its positional demands to the patient in the early postoperative make results dependent not only on the surgical success but to the patient adherence to the recommendations, additionally complications such as: visual disturbances, high intraocular pressure and migration to the anterior chamber are also present with other tamponades different to PFC.

## Conclusion

This scoping review of the literature presents the results of case series and cohorts. However, research with more rigorous methodologies is required to determine the true effect of PFC as a temporary tamponade used in vitreoretinal surgery in terms of visual and structural outcomes and potential risks.

### Electronic supplementary material

Below is the link to the electronic supplementary material.

Supplementary Material 1

Supplementary Material 2

## Data Availability

All data generated or analyzed during this study are included in this article. Further enquiries can be directed to the corresponding author.

## References

[1] Yu H, Li J, Yu Y, Li G, Li D, Guan M (2019). Optimal timing of vitrectomy for severe mechanical ocular trauma: a retrospective observational study. Sci Rep Diciembre De.

[2] Schrader WF. Open Globe Injuries: Epidemiological Study of Two Eye Clinics in Germany, 1981–1999. Croat Med J.:7. 15185415

[3] Georgalas I, Ladas I, Tservakis I, Taliantzis S, Gotzaridis E, Papaconstantinou D (2011). Perfluorocarbon liquids in vitreoretinal Surgery: a review of applications and toxicity. Cutan Ocular Toxicol Diciembre De.

[4] Chang S, Reppucci V, Zimmerman NJ, Heinemann M-H, Coleman DJ (1989). Perfluorocarbon liquids in the management of traumatic retinal detachments. Ophthalmol Junio De.

[5] Pikkel J, Chassid O, Sharabi – Nov A, Beiran I. Short term postoperative tamponade using perfluorocarbon liquid for treatment of giant retinal tears. Int Eye Sci. Oct. 2013;13(10). 10.3980/j.issn.1672-5123.2013.10.01.

[6] Bhardwaj G, Connell PP, Campbell WG (2020). MANAGEMENT OF GIANT RETINAL TEARS USING TRANSSCLERAL DIODE LASER RETINOPEXY AND SHORT-TERM POSTOPERATIVE TAMPONADE WITH PERFLUORO-N-OCTANE. Retina.

[7] Gershoni ME-MA, Axer-Siegel R, Rita Ehrlich (2017). Short-term Vitreoretinal Tamponade with Heavy Liquid following Surgery for Giant Retinal tear. Curr Eye Res.

[8] Ventura MC, Melo C, Escarião P, Diniz JR, Leão AC. Perfluoroctano líquido como tamponante vitreorretiniano de curta duração no pós-operatório de portadores de descolamento de retina por ruptura gigante (Perfluoroctane liquid as a short-term vitreous-retinal tamponade in the postoperative period in patients with retinal detachment due to giant tears). Arq Bras Oftalmol. 2007 May-Jun;70(3):495–500. Portuguese. 10.1590/s0004-27492007000300018. PMID: 17768558. 10.1590/s0004-27492007000300018 17768558

[9] Sirimaharaj M, Balachandran C, Chan WC (2005). Vitrectomy with short term postoperative tamponade using perfluorocarbon liquid for giant retinal tears. Br J Ophthalmol.

[10] Zeana D, Becker J, Kuckelkorn R (1999). Perfluorohexyloctane as a long-term vitreous tamponade in the experimental animal. Int Ophthalmol.

[11] Orzalesi N, Migliavacca L, Bottoni F, Miglior S (1998). Experimental short-term tolerance to perfluorodecalin in the rabbit eye: a histopathological study. Curr Eye Res.

[12] Drury B, Bourke RD (2011). Short-term intraocular tamponade with perfluorocarbon heavy liquid. Br J Ophthalmol.

[13] Rofail M, Lee LR. Perfluoro-n-octane as a postoperative vitreoretinal tamponade in the management of giant retinal tears. Retina. 2005 Oct-Nov;25(7):897–901. 10.1097/00006982-200510000-00013. PMID: 16205570. 10.1097/00006982-200510000-00013 16205570

[14] Chehade LK, Guo B, Chan W, Gilhotra J (2021). Medium-term tamponade with vitrectomy and perfluorodecalin for the management of complex retinal detachments. Eur J Ophthalmol.

[15] Bottoni, F., de Molfetta, V., Monticelli, M., Prussiani, A., Arpa, P., & Bailo, G.(n.d.). Management of giant retinal tears using perfluorodecalin as a … https://journals.healio.com/doi/10.3928/1542-8877-19940601-06. 8090415

[16] Scott IU, Flynn HW, Murray TG, Feuer WJ (2003). Perfluoron study group. Outcomes of Surgery for retinal detachment associated with proliferative vitreoretinopathy using perfluoro-n-octane: a multicenter study. Am J Ophthalmol.

[17] Sigler EJ, Randolph JC, Calzada JI, Charles S (2013). Pars plana vitrectomy with medium-term postoperative perfluoro-N-octane for recurrent inferior retinal detachment complicated by advanced proliferative vitreoretinopathy. Retina.

[18] Bhurayanontachai P, Seepongphun U (2020). Outcomes of a postoperative perfluorocarbon liquid tamponade for complex retinal detachments: 12 years of experience in southern Thailand. BMC Ophthalmol.

[19] Wilkinson CP (2009). Mysteries regarding the surgically reattached retina. Trans Am Ophthalmol Soc.

[20] Rush RMD, Sheth SMD, Surka SMD, Ho IMD, Gregory-Roberts JMD, POSTOPERATIVE PERFLUORO-N-OCTANE TAMPONADE FOR PRIMARY RETINAL DETACHMENT REPAIR. Retina 32(6):p 1114–1120, June 2012. | 10.1097/IAE.0b013e31822f56f6. 10.1097/IAE.0b013e31822f56f6 21968506

[21] Lorenzi U, Mora P, Luciani E, Barale P-O, Muraine M, PERFLUOROCARBON LIQUID BUBBLES COMPLICATING RETINAL DETACHMENT SURGERY USING AIR for DRAINAGE (2022).

[22] Smith AG, Cost BM, Ehlers JP (2015). Intraoperative OCT-assisted subretinal perfluorocarbon liquid removal in the DISCOVER study. Ophthalmic Surg Lasers Imaging Retina.

