# Supplementary material for: Efficacy of the use of perfluorocarbon as a temporary tamponade agent in severe ocular trauma and/or complex retinopexy: a scoping review
Source: Int J Retina Vitreous. 2024 Jan 18;10:6. doi: 10.1186/s40942-023-00504-6 (PMC10797930; doi:10.1186/s40942-023-00504-6)
Supplement: Supplementary file 1 — Supplementary Material 1 [file 40942_2023_504_MOESM1_ESM.docx]

Sara Margarita Pérez Pérez

Street 7 # 40-62.  Bogotá, Colombia

saraperez@javeriana.edu.co

Phone:  +57 (314) 4625772

Ophthalmology Unit, Pontifical Xavierian University

10 October 2023

Dear **International Journal of Retina and Vitreous,**

I am writing on behalf of the co-authors of our research manuscript titled **"Efficacy of Perfluorocarbon as a Temporary Tamponade Agent in Severe Ocular Trauma and Complex Retinopexy: A Scoping Review."** We wish to express our strong interest in having our work published in the esteemed International Journal of Retina and Vitreous.

In response to the invaluable feedback provided by the reviewers and the editorial team, we have dedicated our efforts to thoroughly revising the manuscript. We are pleased to inform you that our manuscript now aligns seamlessly with the high standards and focus of the journal.

With great diligence, we have addressed every comment and suggestion provided during the review process, resulting in a more robust, methodical, and insightful piece of research. These revisions have significantly elevated the clarity and depth of our work, rendering it even more pertinent to the broader medical community.

Thank you for your time and consideration. I have attached the revised manuscript, along with the response to reviewers and any supplementary materials as per your journal's submission guidelines. I look forward to hearing from you regarding the status of my submission.

Should you require any further information or have any questions, please do not hesitate to contact me at **+57 3144625772** or via email at **saraperez@javeriana.edu.co**

Thank you for your dedication to advancing medical knowledge, and I hope to have the opportunity to contribute to the outstanding work of **International Journal of Retina and Vitreous**.

Sincerely,


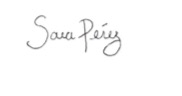


Sara Margarita Pérez Pérez

Ophthalmology Unit, Pontifical Xavierian University
