# Supplementary material for: Efficacy of the use of perfluorocarbon as a temporary tamponade agent in severe ocular trauma and/or complex retinopexy: a scoping review
Source: Int J Retina Vitreous. 2024 Jan 18;10:6. doi: 10.1186/s40942-023-00504-6 (PMC10797930; doi:10.1186/s40942-023-00504-6)
Supplement: Supplementary file 2 — Supplementary Material 2 [file 40942_2023_504_MOESM2_ESM.docx]

**Supplementary material**

**Supplementary Table 2. Search algorithm in MEDLINE and Cochrane Central Register of Controlled Trials.**

| **Electronic search report No.1** | |
| --- | --- |
| **Type of search** | New |
| **Databases** | - MEDLINE - MEDLINE In-Process & Other Non-Indexed Citations - MEDLINE Daily Update - Cochrane Central Register of Controlled Trials -CENTRAL |
| **Platform** | Ovid |
| **Date of search** | 19 September 2022 |
| **Search date range** | Unrestricted |
| **Language restrictions** | none |
| **Other limits** | None |
| **Search strategy (results)** | exp Eye Injuries/  (eye adj3 injury).tw.  exp Eye Injuries, Penetrating/  (Penetrating adj3 Eye adj3 Injur$).tw.  exp retinal detachment/  1 or 2 or 3 or 4 or 5  exp Vitreoretinal Surgery/  (surgery adj3 vitreoretinal).tw.  7 or 8  perfluorocarbon.tw.  6 and 9 and 10 |
| **References identified** | 52 |
| **References without duplicates** | 52 |

**Supplementary Table 3. EMBASE Search Algorithm**

| **Electronic search report No.2** | |
| --- | --- |
| **Type of search** | New |
| **Database** | EMBASE |
| **Platform** | Elsevier |
| **Date of search** | 1 October 2022 |
| **Search date range** | Unrestricted |
| **Language restrictions** | None |
| **Other limits** | EMBASE database only |
| **Search strategy (results)** | 1. 'vitreoretinal surgery'/exp  2. (vitreo-retinal NEAR/3 surgery): ab, ti  3. (vitreoretinal NEAR/3 procedur$): ab, ti  4. #1 OR #2 OR #3  5. 'fluorocarbon'/exp  6. (fluorine NEAR/3 carbon): ab, ti  7. (fluorocarbon NEAR/3 compound): ab, ti  8. (fluorocarbon NEAR/3 derivative): ab, ti  9. (fluorocarbon NEAR/3 extract): ab, ti  10. (fluorocarbon NEAR/3 polyme$): ab, ti  11. (fluorocarbon NEAR/3 solvent): ab, ti  12. (fluorocarbons): ab, ti  13. (Perfluorocarbon): ab, ti  14. (perfluorocarbon NEAR/3 compound): ab, ti  15. #5 OR #6 OR #7 OR #8 OR #8 OR #9 OR #10 OR #11 OR #12 OR #13 OR #14  16. tamponade  17. #4 AND #15 and #16  18. 'short term'.  19. #4 AND #15 and #18 |
| **References identified** | 190 |
| **References without duplicates** | 190 |

**Supplementary Tables 4. List of included studies**

| **Authors** | Bhardwaj, Gaurav; Connell, Paul P; Campbell, William G. |
| --- | --- |
| **Title** | Management of giant retinal tears using transscleral diode laser retinopexy and short-term postoperative tamponade with perfluoro-n-octane |
| **Type of study** | Case series |
| **Year of publication** | 2020 |
| **Population** | Twenty consecutive subjects with recent giant retinal tears |
| **Intervention** | Short-term PFC-tamponadeed pars plana vitrectomy. Only subjects who were followed for 3 months or more were included. |
| **Comparison** | There was none |
| **Outcome** | Retinal reattachment and postoperative complications. |

| **Authors** | [Maya Eiger-Moscovich](https://www.tandfonline.com/author/Eiger-Moscovich%2C+Maya), [Assaf Gershoni](https://www.tandfonline.com/author/Gershoni%2C+Assaf), [Ruth Axer-Siegel](https://www.tandfonline.com/author/Axer-Siegel%2C+Ruth), [Dov Weinberger](https://www.tandfonline.com/author/Weinberger%2C+Dov); [Rita Ehrlich](https://www.tandfonline.com/author/Ehrlich%2C+Rita) |
| --- | --- |
| **Title** | Short-Term Vitreoretinal Tamponade with Heavy Liquid Following Surgery for Giant Retinal Tear |
| **Type of study** | Case series |
| **Year of publication** | 2017 |
| **Population** | 13 subjects (13 eyes) who presented with giant retinal tears (GRT). |
| **Intervention** | Subjects treated with vitrectomy followed by short-term tamponade with heavy perfluorocarbon fluid. A minimum follow-up of 3 months was required for inclusion. |
| **Comparison** | There was none |
| **Outcome** | Retinal reattachment and postoperative complications. |

| **Authors** | Joseph Pikkel, Otzem Chassid, Adi Sharabi-Nov, Itzchak Beiran |
| --- | --- |
| **Title** | Short term postoperative tamponade using perfluorocarbon liquid for treatment of giant retinal tears |
| **Type of study** | Cohort study |
| **Year of publication** | 2013 |
| **Population** | Subjects who underwent surgery for retinal detachment caused by GRT |
| **Intervention** | Short-term postoperative PFC was used for retinal detachment due to GRTs. Subjects treated before January 2002 constituted the control group of this study, and those treated after this date constituted the study group. All surgeries were performed by a single surgeon. |
| **Comparison** | Subjects treated before January 2002 constituted the control group of this study. |
| **Outcome** | Retinal reattachment and postoperative complications. |

| **Authors** | Marcelo Carvalho VenturaI;Cecília MeloII;Paulo EscariãoII; José Ricardo DinizII; Ana Cecília de Souza LeãoI |
| --- | --- |
| **Title** | Perfluoroctane liquid as a short-term vitreous-retinal tamponade in the postoperative period in subjects with retinal detachment due to giant tears. |
| **Type of study** | Case Series |
| **Year of publication** | 2007 |
| **Population** | Ten subjects with rhegmatogenous retinal detachment secondary to GRT complicated by proliferative vitreoretinopathy (PVR) grade B or worse seen in a retina and vitreous department of a referral ophthalmology service between 2000 and 2005. |
| **Intervention** | 1 PPV with vitrectomy probe (20-gauge) and 3 sclerotomies were performed. The Accurus®-Alcon Laboratories vitrectomy device was used. When necessary, the vitreoretinal pick and intraocular forceps were used to remove the preretinal membranes and then PFC was placed as a temporary tamponade. |
| **Comparison** | There was none |
| **Outcome** | Retinal reattachment and postoperative complications. |

| **Authors** | Rofail, Marc; Lee, Lawrence R. |
| --- | --- |
| **Title** | Perfluoro-n-octane as a postoperative vitreoretinal tamponade in the management of giant retinal tears |
| **Type of study** | Case series |
| **Year of publication** | 2005 |
| **Population** | 16 cases of GRTs |
| **Intervention** | Application of perfluoro-n-octane type PFC and performance of vitrectomy plus laser with subsequent removal of the tamponade two weeks after the procedure. |
| **Comparison** | There was none |
| **Outcome** | Retinal reattachment and postoperative complications. |

| **Authors** | M Sirimaharaj**,** C Balachandran**,** W C Chan**,** A P Hunyor**,** A A Chang**,** J G Roberts**,** A B Hunyor, T J Playfair, A A A Chang**,** J G Roberts**,** A B Hunyor**,** T J Playfair |
| --- | --- |
| **Title** | Vitrectomy with short term postoperative tamponade using perfluorocarbon liquid for giant retinal tears |
| **Type of study** | Case series |
| **Year of publication** | 2005 |
| **Population** | 65 subjects with GRTs |
| **Intervention** | Vitrectomy plus endodiathermy was performed to mark the edge of the tears. Subsequently, perfluoro-n-octane was injected as a temporary tamponade with subsequent removal for 5 to 14 days. |
| **Comparison** | There was none |
| **Outcome** | Retinal reattachment and postoperative complications. |

| **Authors** | Daminana Zeana, Jacob Becker, Ralf Kuckelkorn and Bernd Kirchhof. |
| --- | --- |
| **Title** | Perfluorohexyloctane as a long-term vitreous tamponade in the experimental animal |
| **Type of study** | Clinical animal experiment |
| **Year of publication** | 1999 |
| **Population** | 34 rabbits |
| **Intervention** | 34 vitrectomized eyes of pigmented rabbits were subjected to intravitreal injection of 1.0-1.2 ml of perfluorohexyloctane or balanced salt solution. |
| **Comparison** | The animals were randomized into 3 groups: group 1, which sought to evaluate the short-term effects of PFC by withdrawing it at two weeks; groups 2 and 3, long-term effects of PFC by withdrawing it at nine and 14 weeks. These results were compared with the same number of rabbit eyes that underwent vitrectomy and were placed in balanced salt solution. |
| **Outcome** | Retinal reattachment and postoperative complications. |

| **Authors** | Luca Migliavacca,Ferdinando Bottoni & Stefano Miglior |
| --- | --- |
| **Title** | Experimental short-term tolerance to perfluorodecalin in the rabbit eye: A histopathological study. |
| **Type of study** | Clinical animal experiment |
| **Year of publication** | 1998 |
| **Population** | 24 rabbit eyes |
| **Intervention** | Performance of vitrectomy with subsequent injection of 1.4-1.6 cc PFC in 18 rabbits. |
| **Comparison** | Six rabbit eyes that underwent vitrectomy plus injection of balanced salt solution. |
| **Outcome** | Retinal reattachment and postoperative complications. |

| **Authors** | Drury B, Bourke RD |
| --- | --- |
| **Title** | Short term intraocular tamponade with perfluorocarbon heavy liquid |
| **Type of study** | Case series |
| **Year of publication** | 2011 |
| **Population** | Retrospective analysis of 17 eyes |
| **Intervention** | Treatment with with short-term perfluoro-n-octane tamponade for complex inferior retinal pathology, including inferior detachment and PVR. |
| **Comparison** | There was none |
| **Outcome** | Retinal reattachment and postoperative complications. |

| **Authors** | Chehade LK, Guo B, Chan W, Gilhotra J. |
| --- | --- |
| **Title** | Medium-term tamponade with vitrectomy and perfluorodecalin for the management of complex retinal detachments. |
| **Type of study** | Case series |
| **Year of publication** | 2021 |
| **Population** | Medical records of 85 eyes   in the last 6 years presenting with complex retinal detachment |
| **Intervention** | vitrectomy where PFD was used as a medium-term tamponade. |
| **Comparison** | There was none |
| **Outcome** | Retinal reattachment and postoperative complications. |

| **Authors** | Bottoni, F., de Molfetta, V., Monticelli, M., Prussiani, A., Arpa, P., & Bailo, G. |
| --- | --- |
| **Title** | Management of giant retinal tears using perfluorodecalin as a temporary tamponade |
| **Type of study** | Case series |
| **Year of publication** | 2013 |
| **Population** | 11 eyes with giant retinal tears and grade B PVR |
| **Intervention** | Lensectomy, vitrectomy, 5 day tamponade with perfluorodecalin (PFD) |
| **Comparison** | There was none |
| **Outcome** | Retinal reattachment and postoperative complications. |

| **Authors** | Rush R, Sheth S, Surka S, Ho I, Gregory-Roberts J |
| --- | --- |
| **Title** | Postoperative perfluoro-N-octane tamponade for primary retinal detachment repair. |
| **Type of study** | Case series |
| **Year of publication** | 2012 |
| **Population** | 39 eyes |
| **Intervention** | primary PFO retention and secondary replacement |
| **Comparison** | There was none |
| **Outcome** | Retinal reattachment and postoperative complications. |

**Supplementary Table 5. List of studies excluded from the full-text assessment and reasons for exclusion.**

| **Authors** | **Year** | **Title** | **Reason for exclusion** |
| --- | --- | --- | --- |
| [Mario R. Romano](https://www.nature.com/articles/s41433-021-01596-w#auth-Mario_R_-Romano), Mariantonia Ferrara, [Irene Nepita](https://www.nature.com/articles/s41433-021-01596-w#auth-Irene-Nepita), [Jana D'Amato Tothova](https://www.nature.com/articles/s41433-021-01596-w#auth-Jana-D_Amato_Tothova), [Alberto Giacometti Schieroni](https://www.nature.com/articles/s41433-021-01596-w#auth-Alberto-Giacometti_Schieroni), [Daniela Reami](https://www.nature.com/articles/s41433-021-01596-w#auth-Daniela-Reami), [Raniero Mendichi](https://www.nature.com/articles/s41433-021-01596-w#auth-Raniero-Mendichi), [Libero Liggieri](https://www.nature.com/articles/s41433-021-01596-w#auth-Libero-Liggieri) & [Rodolfo Repetto](https://www.nature.com/articles/s41433-021-01596-w#auth-Rodolfo-Repetto) | 2021 | Biocompatibility of intraocular liquid tamponade agents: an update | Review of the literature describing tamponades but not their use as a temporary tamponade |
| Paolo Chelazzi, Claudia Azzolini, Claudia Bellina,Francesca Cappelli,Ilaria Del Genovese Laura Caraffa and Francesco Scullica | 2021 | Efficacy and Safety of Vitrectomy without Using Perfluorocarbon Liquids and Drainage Retinotomy Associated with Postoperative Positioning Based on Residual Subretinal Fluid for Rhegmatogenous Retinal Detachment | Does not meet inclusion criteria |
| [Eduardo B Rodrigues](https://pubmed.ncbi.nlm.nih.gov/?term=Rodrigues+EB&cauthor_id=24480841) [1,](https://pubmed.ncbi.nlm.nih.gov/24480841/#affiliation-1) [Helio Shiroma](https://pubmed.ncbi.nlm.nih.gov/?term=Shiroma+H&cauthor_id=24480841), [Fernando M Penha](https://pubmed.ncbi.nlm.nih.gov/?term=Penha+FM&cauthor_id=24480841), [Mauricio Maia](https://pubmed.ncbi.nlm.nih.gov/?term=Maia+M&cauthor_id=24480841), [Milton N Moraes-Filho](https://pubmed.ncbi.nlm.nih.gov/?term=Moraes-Filho+MN&cauthor_id=24480841), [Magno Ferreira](https://pubmed.ncbi.nlm.nih.gov/?term=Ferreira+M&cauthor_id=24480841), [Renata Portella](https://pubmed.ncbi.nlm.nih.gov/?term=Portella+R&cauthor_id=24480841), [Eduardo Novais](https://pubmed.ncbi.nlm.nih.gov/?term=Novais+E&cauthor_id=24480841), [Nadine Hagedorn](https://pubmed.ncbi.nlm.nih.gov/?term=Hagedorn+N&cauthor_id=24480841), [Michel E Farah](https://pubmed.ncbi.nlm.nih.gov/?term=Farah+ME&cauthor_id=24480841) | 2014 | Development and initial experience with a colored perfluorocarbon liquid for intraocular tamponade in vitreoretinal surgery | Does not meet inclusion criteria |
| [V S Gurunadh](https://pubmed.ncbi.nlm.nih.gov/?term=Gurunadh+VS&cauthor_id=27375321), [A Banarji](https://pubmed.ncbi.nlm.nih.gov/?term=Banarji+A&cauthor_id=27375321), [S Patyal](https://pubmed.ncbi.nlm.nih.gov/?term=Patyal+S&cauthor_id=27375321), [A K Upadhyay](https://pubmed.ncbi.nlm.nih.gov/?term=Upadhyay+AK&cauthor_id=27375321), [T S Ahluwalia](https://pubmed.ncbi.nlm.nih.gov/?term=Ahluwalia+TS&cauthor_id=27375321), [R P Gupta](https://pubmed.ncbi.nlm.nih.gov/?term=Gupta+RP&cauthor_id=27375321), [M Bhaduria](https://pubmed.ncbi.nlm.nih.gov/?term=Bhaduria+M&cauthor_id=27375321) | 2010 | Evaluation of vitreous substitutes in managing complicated vitreo-retinal surgeries. | Does not meet inclusion criteria |
| Timothy L.JacksonPhD, FRCOphthAnthony S.L.KwanMD, FRCOphthAlistair H.LaidlawMD, FRCOphthWilliam Aylward, FRCOphth | 1991 | Identification of Retinal Breaks Using Subretinal Trypan Blue Injection | Type of intervention |
| [Aude Ambresin](https://pubmed.ncbi.nlm.nih.gov/?term=Ambresin+A&cauthor_id=14574245) [1](https://pubmed.ncbi.nlm.nih.gov/14574245/#affiliation-1), [Thomas J Wolfensberger](https://pubmed.ncbi.nlm.nih.gov/?term=Wolfensberger+TJ&cauthor_id=14574245), [Etienne H Bovey](https://pubmed.ncbi.nlm.nih.gov/?term=Bovey+EH&cauthor_id=14574245) | 2003 | Management of giant retinal tears with vitrectomy, internal tamponade, and peripheral 360° retinal photocoagulation | Does not meet inclusion criteria |
